# Metagenomic analysis of microbial consortium from natural crude oil that seeps into the marine ecosystem offshore Southern California

**DOI:** 10.4056/sigs.5029016

**Published:** 2014-01-02

**Authors:** Erik R. Hawley, Hailan Piao, Nicole M. Scott, Stephanie Malfatti, Ioanna Pagani, Marcel Huntemann, Amy Chen, Tijana Glavina del Rio, Brian Foster, Alex Copeland, Janet Jansson, Amrita Pati, Susannah Tringe, Jack A. Gilbert, Thomas D. Lorenson, Matthias Hess

**Affiliations:** 1 Washington State University Tri-Cities, Richland, WA, USA; 2 Argonne National Laboratory, Lemont, IL, USA; 3 Lawrence Livermore National Laboratory, Biosciences and Biotechnology Division, Livermore, CA, USA; 4 DOE Joint Genome Institute, Walnut Creek, CA, USA; 5 Lawrence Berkeley National Laboratory, Berkeley, CA, USA; 6 University of Chicago, Chicago, IL, USA; 7 USGS, Menlo Park, CA, USA; 8 Washington State University, Pullman, WA, USA; 9 Pacific Northwest National Laboratory, Chemical & Biological Process Development Group, Richland, WA, USA; 10 Environmental Molecular Sciences Laboratory, Richland, WA, USA

**Keywords:** Bioremediation, hydrocarbon-degradation, marine ecosystem, crude oil, natural oil seeps, anaerobic methane oxidation, bacteria, archaea, metagenomics

## Abstract

Crude oils can be major contaminants of the marine ecosystem and microorganisms play a significant role in the degradation of its main constituents. To increase our understanding of the microbial hydrocarbon degradation process in the marine ecosystem, we collected crude oil from an active seep area located in the Santa Barbara Channel (SBC) and generated a total of about 52 Gb of raw metagenomic sequence data. The assembled data comprised ~500 Mb, representing ~1.1 million genes derived primarily from chemolithoautotrophic bacteria. Members of *Oceanospirillales*, a bacterial order belonging to the *Deltaproteobacteria*, recruited less than 2% of the assembled genes within the SBC metagenome. In contrast, the microbial community associated with the oil plume that developed in the aftermath of the Deepwater Horizon (DWH) blowout in 2010, was dominated by *Oceanospirillales*, which comprised more than 60% of the metagenomic data generated from the DWH oil plume. This suggests that *Oceanospirillales* might play a less significant role in the microbially mediated hydrocarbon conversion within the SBC seep oil compared to the DWH plume oil. We hypothesize that this difference results from the SBC oil seep being mostly anaerobic, while the DWH oil plume is aerobic. Within the *Archaea*, the phylum *Euryarchaeota*, recruited more than 95% of the assembled archaeal sequences from the SBC oil seep metagenome, with more than 50% of the sequences assigned to members of the orders *Methanomicrobiales* and *Methanosarcinales*. These orders contain organisms capable of anaerobic methanogenesis and methane oxidation (AOM) and we hypothesize that these orders – and their metabolic capabilities – may be fundamental to the ecology of the SBC oil seep.

## Introduction

Oil-exposed marine microbial consortia are known to be capable of degrading hydrocarbons [1]. Hydrocarbon-degrading microbes have been used successfully in the remediation of oil that contaminated long stretches of shorelines [2,3]; and it was endorsed anew as a promising remediation strategy after the Deepwater Horizon (DWH) blowout [4]. Despite the significant resources that have been spent to study the microbial response to oil spills, most of the research data come from culture-based studies and relatively little is known about the dynamics and microbial processes that occur during the biological degradation of crude oil in uncontrolled and highly complex biological systems [5-8]. Advances in DNA sequencing technologies and computation provide insights into the metabolic blueprint of microbial cells and microbial communities directly from environmental samples. This has facilitated a better understanding of the genes and metabolic processes that underlie the phenotypes of individual cells and complex communities - without depending on axenic microbial cultures [9,10]. The potential of DNA sequencing to improve our understanding of microbial responses to large oil spills, was recognized immediately by the scientific community following the 4 million barrel DWH spill released into the Gulf of Mexico (GoM), resulting in a number of studies that employed metagenomics and metatranscriptomics to map the communities genetic response so as to eventually develop more sustainable remediation strategies [4,11-14]. The GoM has many natural oil seeps, which have primed the microbial community to be ready for larger spills. As the composition of the natural microbial community at a spill site could have a significant role in the bioremediation process following an oil spill [15], and considering that oil spills are not restricted to the GoM, it will be crucial to build an extended knowledgebase of native hydrocarbon degrading microbiomes from different geographical locations. Here we report on the first metagenome exceeding 50 Gb of raw DNA sequence data from a microbial community associated with natural crude oil seeps of the Santa Barbara Channel (SBC), one of the world’s largest natural hydrocarbon seep regions [16], which can be accessed publicly through IMG/M for further analysis by the scientific community.

## Classification and features

A metagenome was generated from a hydrocarbon-adapted consortium collected using a remotely operated vehicle from a submarine oil seep located near Coal Oil Point at 34.39192° N, 119.84578° W, 79.4 m below sea level [Table 1]. The collected oil samples were transported immediately to the laboratory and stored at -20°C until DNA extraction was performed. Further details of sampling location and oil geochemistry have been described previously by Lorenson and colleagues [19].

**Table 1:** Classification and general features of the metagenome data set according to the Minimum Information about Genomes and Metagenomes (MIMS) standards [17].

**MIMS ID**	**Property**	**Term**	**Evidence code**^a^
MIM 3	Study Name	Marine microbial communities from the Santa Barbara Channel oil seeps	
	Sample Name	Crude oil metagenome 2	
	GOLD classification: Ecosystem	Environmental	NAS
	GOLD classification: Ecosystem Category	Aquatic	
	GOLD classification: Ecosystem Type	Marine	
	GOLD classification: Ecosystem Subtype	Oil seeps	
	GOLD classification: Specific Ecosystem	unclassified	
MIGS-22	Carbon source	Seep oil	NAS
	Energy source	Seep oil	NAS
MIGS-6	Habitat	Aquatic, Marine, Oil seeps	NAS
MIGS-14	Pathogenicity	none	NAS
MIGS-4	Geographic location	Marine ecosystem, California, USA	NAS
MIGS-5	Sample collection time	June, 2009	NAS
MIGS-4.1	Latitude	34.39192	NAS
MIGS-4.2	Longitude	−119.84578	NAS
MIGS-4.3	Depth	79.4 m	NAS

^a^Evidence codes - NAS: Non-traceable Author Statement (i.e. not directly observed for the living, isolated sample, but based on a generally accepted property for the species, or anecdotal evidence). These evidence codes are from the Gene Ontology project [18].

## 

## Metagenome sequencing information

### Metagenome project history

This is the first metagenome associated with natural crude oils that seep into the SBC. The site was selected based on its geographical location near active offshore drilling and the distinct geochemical composition of SBC seep oils compared to those from the GoM. Sequence analysis of small subunit ribosomal RNA gene amplicons identified 1,045 taxa based on 97% sequence identity, and a fingerprint that is distinct from the community associated with the oil plume that formed after the DWH accident [20].

### Growth conditions and DNA isolation

Environmental DNA (eDNA) was extracted from the seep oil sample using a FastDNA Spin Kit for Soil from MP Biomedicals according to the manufacturer’s protocol with 500mg of the seep oil as starting material. Bead-beating was conducted three times for 20 seconds using a Mini-Beadbeater-16 (Biospec Products, Bartlesville, OK, USA). Samples were kept on ice for 1 min between each round of bead-beating. Extracted eDNA was resuspended in a total of 100µL DNase/Pyrogen-Free H_2_0. Concentration of obtained eDNA was measured using a Qubit 2.0 Fluorometer (Life Technologies, Grand Island, NY) according to the manufacturer's protocol. The quantity and quality of the extraction were checked by gel electrophoresis using standards for standard operational procedures of the Joint Genome Institute (JGI).

## Metagenome sequencing and assembly

A total of 51.7 Gbp were generated from the oil-associated microbiome. Starting material (200ng of DNA) was sheared to 270 bp using the Covaris E210 (Covaris) and size selected using SPRI beads (Beckman Coulter). The fragments were treated with end-repair, A-tailing, and ligation of Illumina compatible adapters (IDT, Inc) using the KAPA-Illumina library creation kit (KAPA Biosystems). The prepared sample libraries were quantified by qPCR using KAPA Biosystem’s next-generation sequencing library qPCR kit and run on a Roche LightCycler 480 real-time PCR instrument. The quantified sample libraries were then prepared for sequencing on the Illumina HiSeq2000 sequencing platform, utilizing a TruSeq paired-end cluster kit, v3, and Illumina’s cBot instrument to generate clustered flowcells for sequencing. Sequencing of the flowcells was performed on the Illumina HiSeq2000 platform using a TruSeq SBS sequencing kit 200 cycles, v3, following a 2x150 indexed run recipe. Raw metagenomic reads were trimmed using a minimum quality score cutoff of 10. Trimmed, paired-end Illumina reads were assembled using SOAPdenovo v1.05 [21] with a range of Kmers (81,85,89,93,97,101). Default settings for all SOAPdenovo assemblies were used (flags: –d 1 and –R). Contigs generated by each assembly (6 total contig sets) were sorted into two pools based on length. Contigs smaller than 1,800 bp were assembled using Newbler (Life Technologies, Carlsbad, CA) in an attempt to generate larger contigs (flags: -tr, -rip, -mi 98, -ml 80). All assembled contigs larger than 1,800 bp, as well as the contigs generated from the final Newbler run, were combined using minimus 2 (flags: -D MINID=98 -D OVERLAP=80) [AMOS [22]] Read depth estimations were based on mapping of the trimmed, screened, paired-end Illumina reads to assembled contigs with BWA [23]. The un-assembled, paired reads were merged with FLASH [24]. The assembled contigs along with the merged, un-assembled reads were submitted to IMG/M for functional annotation. Sequences are publicly available at IMG/M under the project ID 45292. Table 2 summarizes the project information and its association with MIGS version 2.0 compliance [17].

**Table 2:** Project information

**MIGS ID**	**Property**	**Term**
MIGS-31	Finishing quality	Standard Draft
MIGS-28	Libraries used	Illumina standard paired-end library (0.27 kb insert size)
MIGS-29	Sequencing platforms	Illumina HiSeq2000
MIGS-31.2	Fold coverage	NA
MIGS-30	Assemblers	SOAPdenovo v1.05, Newbler v2.5, minimus2
MIGS-32	Gene calling method	Genemark > Prodigal > Metagene > FragGeneScan
	GOLD ID	Gm0045292
	GOLD sample ID	Gs0002474
	IMG Project ID	45292
	Project relevance	biodegradation of pollutants, biotechnological

### Metagenome annotation

Prior to annotation, all sequences were trimmed to remove low quality regions falling below a minimum quality of Q13, and stretches of undetermined sequences at the ends of contigs were removed. Each sequence was checked with the DUST algorithm [25] from the NCBI toolkit for low complexity regions and sequences with less than 80 unmasked nt were removed. Additionally very similar sequences (similarity > 95%) with identical 5’ pentanucleotides are replaced by one representative, typically the longest, using uclust [26]. The feature prediction pipeline included the detection of non-coding RNA genes (tRNA, and rRNA), followed by prediction of protein coding genes. Identification of tRNAs was performed using tRNAScan-SE-1.23 [27]. In case of conflicting predictions, the best scoring predictions were selected. Since the program cannot detect fragmented tRNAs at the end of the sequences, we also checked the last 150 nt of the sequences by comparing these to a database of nt sequences of tRNAs identified in the isolate genomes using blastn [28]. Hits with high similarity were kept; all other parameters are set to default values. Ribosomal RNA genes (tsu, ssu, lsu) were predicted using the hmmsearch [29] with internally developed models for the three types of RNAs for the domains of life.

Identification of protein-coding genes was performed using four different gene calling tools, GeneMark (v.2.6r) [30], Metagene (v. Aug08) [31], Prodigal (v2.50) [32] and FragGeneScan [33] all of which are *ab initio* gene prediction programs. We typically followed a majority rule based decision scheme to select the gene calls. When there was a tie, we selected genes based on an order of gene callers determined by runs on simulated metagenomic datasets (Genemark > Prodigal > Metagene > FragGeneScan). At the last step, CDS and other feature predictions were consolidated. The regions identified previously as RNA genes were preferred over protein-coding genes. Functional prediction followed and involved comparison of predicted protein sequences to the public IMG database (db) using the usearch algorithm [26], the COG db using the NCBI developed PSSMs [34], and the pfam db [35] using hmmsearch. Assignment to KEGG Ortholog protein families was performed using the algorithm described in [36].

## Metagenome properties

The metagenome presented here contains 333,405,037 high-quality reads, totaling 50,010,755,550 bp. 38.80% of the reads were assembled into a total of 803,203 scaffolds, representing 495,862,225 bp, with 91,522 scaffolds ≥1 kb, 1,354 scaffolds ≥10 kb, 103 scaffolds ≥25 kb, 6 scaffolds ≥50 kb and 1 scaffold ≥250 kb. The GC content of the assembled metagenome was 44.95%, which is slightly higher compared to the 40.95% observed for the assembled metagenome from the oil plume (IMG ID 1892) that formed in the GoM after the DWH blowout in 2010 [14].

The assembled sequences included 1,143,552 predicted genes with 99.32% annotated as protein-coding genes. A total of 770,455 of the protein coding genes, corresponding to 67.37% of the total predicted protein-coding genes, were assigned to a putative family or function based on the presence of conserved Pfam domains with the remaining genes annotated as hypothetical proteins. The properties and the statistics of the metagenome are summarized in Table 3.

**Table 3:** Nucleotide content and gene count levels of the assembled SBC oil seep metagenome

**Attribute**	**Value**	**% of Total**
Total base pairs sequenced (Gb)	51.7	%100
Total number of sequences (scaffolds)	803,203	38.80%
DNA, total number of bases	495,862,225	0.99%
DNA G+C number of bases	222,883,192	44.95%*
Genes		
RNA genes	7,742	0.68%
rRNA genes	1,827	0.16%
5S rRNA	420	0.04%
16S rRNA	520	0.05%
18S rRNA	12	0.00%
23S rRNA	866	0.08%
28S rRNA	9	0.00%
tRNA genes	5,915	0.52%
Protein coding genes	1,135,810	99.32%
with Product Name	617,327	53.98%
with COG	620,853	54.29%
with Pfam	770,455	67.37%
with KO	461,840	40.39%
with Enzyme	265,509	23.22%
with MetaCyc	182,179	15.93%
with KEGG	266,160	23.27%
COG Clusters	4724	96.94%
Pfam Clusters	14,501	97.77%

* GC percentage shown as count of G's and C's divided by a total number of G's, C's, A's, and T's. This is not necessarily synonymous with the total number of bases.

From the 1,135,810 genes predicted to encode proteins, 620,853 (54.66%) were assigned to one of the 25 general COG categories [Table 4]. Within genes for which a function could be assigned, most genes were assigned to COG categories (E) and (C), which are associated with amino acid transport and energy production and conversion respectively.

**Table 4:** Percentage of genes associated with the 25 general COG functional categories in two assembled metagenomes from hydrocarbon-enriched environments

**Code**	**%age**	**Description**
J	5.71	Translation, ribosomal structure and biogenesis
A	0.06	RNA processing and modification
K	5.41	Transcription
L	6.3	Replication, recombination and repair
B	0.08	Chromatin structure and dynamics
D	1.1	Cell cycle control, cell division, chromosome partitioning
Y	<0.01	Nuclear structure
V	2.13	Defense mechanisms
T	5.54	Signal transduction mechanisms
M	6.28	Cell wall/membrane/envelope biogenesis
N	1.31	Cell motility
Z	0.02	Cytoskeleton
W	<0.01	Extracellular structures
U	2.34	Intracellular trafficking, secretion, and vesicular transport
O	4.12	Posttranslational modification, protein turnover, chaperones
C	8.16	Energy production and conversion
G	5.16	Carbohydrate transport and metabolism
E	8.82	Amino acid transport and metabolism
F	2.66	Nucleotide transport and metabolism
H	4.2	Coenzyme transport and metabolism
I	3.6	Lipid transport and metabolism
P	5.05	Inorganic ion transport and metabolism
Q	1.88	Secondary metabolites biosynthesis, transport and catabolism
R	12.12	General function prediction only
S	7.95	Function unknown

## Taxonomic gene diversity

The taxonomic diversity and phylogenetic structure of the oil metagenome were determined based on the assembled genes, classifying at a minimum 60% identity to members of the listed phyla. The phylogeny reported is the one used in IMG/M [37], which uses the phylogeny described as part of the *Genomic Encyclopedia of* *Bacteria* *and* *Archaea* (GEBA) project [38].

After removing sequences that could not be assigned phylogenetically, the assembled SBC oil seep metagenome was dominated by prokaryotic genes, with the *Proteobacteria*, *Firmicutes*, *Bacteroidetes* and *Chloroflexi* recruiting 12.9%, 6.5%, 2.3% and 2%, respectively, of the 1,135,810 protein encoding sequences with a phylogenetic classification. With 6,380 sequences, the archaeal phylum *Euryarchaeota*, recruited the fifth most sequences, suggesting that this phylum contributes to a large fraction of the microbial sequence data generated from the SBC seep oil. From the genes assigned to the *Proteobacteria*, genes assigned to *Deltaproteobacteria*, *Epsilonproteobacteria*, and *Gammaproteobacteria* were approximately equally frequent in the metagenome, recruiting about 15.8%, 15.2% and 12.4%, respectively, of the 294,783 genes classified as being of bacterial origin. Within the *Deltaproteobacteria*, 54% of the genes categorized at the family level were assigned to strains belonging to the sulfur-reducing *Desulfobacteraceae* (contributing 49%) and *Desulfobulbaceae* (contributing 15%) – bacterial families frequently found associated with hydrocarbon-rich sediments [39-42]. From the genes assigned to the *Epsilonproteobacteria*, only ~14% could be assigned at the family level within the *Helicobacteraceae* and *Campylobacteraceae*, phylogenetic groups that contain several well-characterized sulfur-oxidizers isolated from marine sediments and underground crude oil storage facilities [43-47], recruiting 68% and 32% of the genes, respectively. The *Gammaproteobacteria* was the most diverse class with the mostly anaerobic or micro-aerobic representatives from the *Chromatiaceae*, *Ectothiorhodospiraceae*, *Methylococcaceae* and *Thiotrichaceae*, recruiting 21%, 11%, 13%, and 12% of the genes that could be assigned at family level. In contrast, the metagenome from the aerobic DWH oil plume was dominated by reads derived the *Oceanospirillales* (~60%)*,* an order of the *Gammaproteobacteria* [14]. Within the SBC metagenome only ~2% of the genes assigned at the family level were recruited by *Oceanospirillales* (i.e. *Bermanella marisrubri*, *Marinomonas mediterranea*, *Marinomonas posidonica* and *Neptuniibacter caesariensis*), suggesting that the metabolic capacities of these strict aerobes might contribute only little to the functionality of the indigenous microbiome associated with the SBC seep oils. There were very few sequences attributed to *Eukaryota*, with representatives from the *Ascomycota*, *Streptophyta*, *Cnidaria*, *Chlorophyta* and *Porifera*, accounting for <0.1% of the sequences. Plasmid population-associated genes were dominated by those associated with *Firmicutes* and *Proteobacteria*, outnumbering double-stranded DNA viruses by about two to one. The taxonomic diversity of the genes assembled from the consortium associated with SBC seep oil is summarized in Table 5. A more detailed analysis of the functional gene diversity of the SBC metagenome can be performed readily through IMG/M.

**Table 5:** Overview of taxonomic gene diversity in the assembled SBC oil seep metagenome.

**Domain**	**Phylum**	**% Hits**
Archaea		
	*Euryarchaeota*	0.56
	*Crenarchaeota*	0.01
	*Thaumarchaeota*	0.01
		
Bacteria		
	*Proteobacteria*	12.88
	*Firmicutes*	6.48
	*Bacteroidetes*	2.33
	*Chloroflexi*	2.01
	*Actinobacteria*	0.48
	*Cyanobacteria*	0.34
	*Ignavibacteria*	0.30
	unclassified	0.20
	*Acidobacteria*	0.13
	*Verrucomicrobia*	0.12
	*Planctomycetes*	0.10
	*Deinococcus-Thermus*	0.10
	*Chlorobi*	0.09
	*Spirochaetes*	0.08
	*Synergistetes*	0.04
	*Thermotogae*	0.04
	*Deferribacteres*	0.04
	*Aquificae*	0.04
	*Nitrospirae*	0.03
	*Fusobacteria*	0.03
	*Thermodesulfobacteria*	0.02
	*Poribacteria*	0.02
	*Lentisphaerae*	0.01
	*Dictyoglomi*	0.01
	*Gemmatimonadetes*	0.01
	*Tenericutes*	0.01
	*Chlamydiae*	0.01
		
Eukarya		
	*Ascomycota*	0.01
	*Streptophyta*	0.01
	*Cnidaria*	0.01
	*Chlorophyta*	0.01
	*Porifera*	00.1
	unclassified	0.01
		
Unassigned		73.38

Although gene counts of representative phyla and classes suggest phylogenetic differences, it can be assumed that the results are biased towards groups whose genomes and marker genes (e.g. 16S and 18S rRNA genes) are overrepresented in genomic reference databases. While the relative abundances of between-phyla comparisons may be questionable based on differential representation in the database, the relative abundances of taxa within a phylum is reflective of the distinct metabolic conditions within an analyzed metagenome [11].

### Functional genes related to methane metabolism

Natural hydrocarbon seeps represent a habitat for microbial communities that might provide the molecular tool kit for sustainable strategies to reduce the negative impact of oil spills. They also are a persistent source of methane (CH_4_) [16], a greenhouse gas whose climate warming potential is 25 times greater than that of CO_2_ [48]. Biological CH_4_ oxidation in the marine ecosystem has been well documented and identified as a CH_4_ sink of global significance [49-51]. Anaerobic oxidation of methane (AOM), mediated by microbiomes associated with ocean sediments and deposits, has been proposed as the dominant biological process responsible for the removal of >300 Tg CH_4_ per year from the ocean [52,53]. Despite strong research efforts aimed at understanding AOM and its regulation, it remains poorly understood. Until recently, AOM in marine environments was thought to be mediated by consortia of anaerobic methanotrophic archaea (ANMEs) and sulfate reducing bacteria [54,55] or alternatively by microbial consortia that couple methane oxidation to the reduction of reactive metals [56]. It was not until 2010 that the first microorganism, Candidatus *Methoxymirabilis oxyfera*, capable of performing methane oxidation (coupled to nitrite reduction) in the absence of a metabolic partner was reported [57], followed by a second organism capable of performing single-organism AOM coupled sulfate reduction [58]. To explore if the indigenous microbial community in the SBC might have the genomic capacity to perform AOM and function as an efficient biofilter when large amounts of methane are released from the ocean subsurface, we generated a profile for genes involved in methane oxidation and methane generation. Pathway analysis based on the KEGG pathways map and the classification systems of the KEGG pathways database, was performed using the “Function Profile” tool implemented in IMG/M. Table 6 summarizes the results of the performed gene profile analysis. Key genes for AOM (and methanogenesis), including genes for the oxygen sensitive formylmethanofuran dehydrogenases (*fmd*; KEGG Orthology IDs K00200, K00201, K00202, K00203, K00205, K11261) and methyl coenzyme M reductases (*mcr*; KEGG Orthology IDs K00399, K00401, K00402) that catalyze the initial and terminal step of methane production, were identified within the metagenome (Table 6). The presence of the key enzymes for AOM would certainly facilitate reversed methanogenesis in an environment that is rich in non-biotic methane by members of the anaerobic methanotrophic *Archaea* (ANME) – as proposed previously by several groups [59,60]. ANME-mediated AOM would explain the dominance of genes from the *Methanomicrobiales* (containing ANME-1) and *Methanosarcinaceae* (containing ANME-2 and ANME-3) [61] within the archaeal genes of the SBC seep oil metagenome (totaling ~56% of the archaeal genes). Active aerobic methane oxidation is restricted to a thin surface layer of seep sediments due to a limited oxygen penetration of less than 2 cm [62]; genes encoding methane monooxygenase (*pmo;* KEGG Orthology IDs K10944, K10945, K10946), a key enzyme of the aerobic methane oxidation process, were identified within the SBC seep oil metagenome (Table 6), suggesting the potential for aerobic methane oxidation. This finding correlates with the fact that members of the *Methylococcaceae,* a group of microorganisms well known for the ability to perform aerobic methane oxidation, comprised ~0.31% of protein coding genes of the SBC seep oil metagenome. This is not the first time that simultaneous evidence of anaerobic and aerobic pathways for methane oxidation in SBC sediments has been reported based on metagenomic data. In 2011, Havelsrud [63] identified the complete suite of key enzymes for AOM in a metagenome from deep sediments (10 - 15 cm) offshore Coal Oil Point in the SBC, whereas sequencing of the shallower sediments (0 - 4 cm) failed to detect two of the key enzymes (methenyl-tetrahydromethanopterin cyclohydrolase and methylenetetrahydromethanopterin dehydrogenase) of AOM. Genes annotated as methane monooxygenase were identified within the shallow sediment metagenome [63], suggesting the possibility that the upper sediment layers of SBC sediments contain pockets of aerobic and anaerobic microhabitats.

**Table 6:** Counts of genes associated with methane metabolism in SBC seep oil metagenome

**KEGG Orthology ID**	**Description**	**Gene count**
K00192	Acetyl-CoA pathway	21
K00195	Acetyl-CoA pathway	6
K00440	Coenzyme F420 hydrogenase	1
K00441	Coenzyme F420 hydrogenase	62
K00443	Coenzyme F420 hydrogenase	3
K05884	Coenzyme M biosynthesis	11
K05979	Coenzyme M biosynthesis	20
K06034	Coenzyme M biosynthesis	2
K08097	Coenzyme M biosynthesis	13
K13039	Coenzyme M biosynthesis	5
K11212	F420 biosynthesis	63
K11780	F420 biosynthesis	7
K11781	F420 biosynthesis	6
K12234	F420 biosynthesis	66
K14941	F420 biosynthesis	40
K00018	Formaldehyde assimilation	77
K00024	Formaldehyde assimilation	277
K00600	Formaldehyde assimilation	463
K00830	Formaldehyde assimilation	116
K00850	Formaldehyde assimilation	558
K00863	Formaldehyde assimilation	2
K01595	Formaldehyde assimilation	133
K01624	Formaldehyde assimilation	276
K01689	Formaldehyde assimilation	380
K03841	Formaldehyde assimilation	122
K08093	Formaldehyde assimilation	20
K08094	Formaldehyde assimilation	32
K08691	Formaldehyde assimilation	35
K08692	Formaldehyde assimilation	13
K11529	Formaldehyde assimilation	6
K13812	Formaldehyde assimilation	14
K13831	Formaldehyde assimilation	26
K14067	Formaldehyde assimilation	14
K16370	Formaldehyde assimilation	10
K16158	Methane oxidation	2
K10944	Methane oxidation; Nitrification	3
K10945	Methane oxidation; Nitrification	3
K10946	Methane oxidation; Nitrification	19
K00200	Methanogenesis	20
K00201	Methanogenesis	27
K00202	Methanogenesis	26
K00203	Methanogenesis	8
K00204	Methanogenesis	0
K00205	Methanogenesis	10
K00319	Methanogenesis	5
K00320	Methanogenesis	111
K00399	Methanogenesis	10
K00401	Methanogenesis	7
K00402	Methanogenesis	3
K00577	Methanogenesis	12
K00578	Methanogenesis	3
K00579	Methanogenesis	7
K00580	Methanogenesis	7
K00581	Methanogenesis	9
K00582	Methanogenesis	2
K00583	Methanogenesis	5
K00584	Methanogenesis	18
K00625	Methanogenesis	77
K00672	Methanogenesis	14
K00925	Methanogenesis	144
K01499	Methanogenesis	21
K01895	Methanogenesis	671
K03388	Methanogenesis	1620
K03389	Methanogenesis	234
K03390	Methanogenesis	137
K04480	Methanogenesis	1
K11260	Methanogenesis	6
K11261	Methanogenesis	67
K13788	Methanogenesis	88
K14080	Methanogenesis	3
K14081	Methanogenesis	1
K14082	Methanogenesis	10
K14083	Methanogenesis	638
K14084	Methanogenesis	56
K16176	Methanogenesis	50
K16177	Methanogenesis	3
K16178	Methanogenesis	9
K16179	Methanogenesis	9
K00193	Methanogenesis; Acetyl-CoA pathway	16
K00194	Methanogenesis; Acetyl-CoA pathway	84
K00197	Methanogenesis; Acetyl-CoA pathway	149

**Figure 1:**
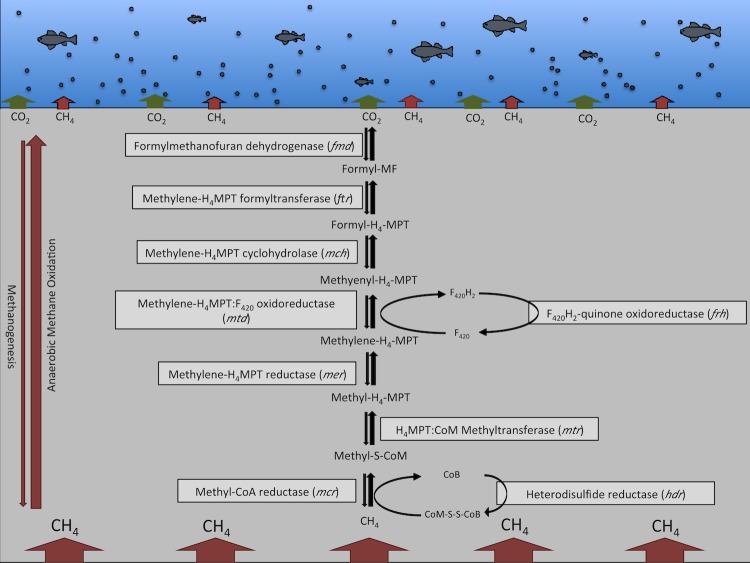
Anaerobic methane oxidation/methanogenesis in sediments of the Santa Barbara Channel. Proposed pathway based on the genes involved in AOM and methanogenesis identified in the metagenome from Santa Barbara Channel seep oil.

To investigate the presence of genomic material from sulfur-reducing bacteria (SRB) – microbes mediating reverse methanogenesis – we analyzed the metagenomes for genes encoding dissimilatory sulfite reductase (*dsr;* KEGG Orthology IDs K11180, K11181). We identified a total of 204 reads annotated as *dsr* within the SBC seep oil metagenome (data not shown), suggesting that AOM via reverse methanogenesis – a process mediated primarily by consortia of archaeal methane oxidizers and bacterial sulfur reducers – may occur during the microbially mediated biofiltration of CH_4_ in the hydrocarbon rich sediments. The proposed CH_4_ biofiltration process under anaerobic conditions within the SBC sediments is summarized in Figure 1. Analysis of the metagenome data from the SBC revealed a total of 2,373 genes covering the complete suite of enzymes necessary for anaerobic methane oxidation/methanogenesis outlined in Figure 1. In contrast, the DWH oil plume metagenome (accessible through IMG/M) contained only a total of 9 genes (i.e. *fwd*, *hdr* and *mer*) that were assigned to this pathways that has been reported as a characteristic feature for microbiomes associated with anaerobic habitats rich in hydrocarbons [42,64,65].

## Conclusion

Sequencing of eDNA extracted from crude oil that was collected from an active hydrocarbon seep in the Santa Barbara Channel (SBC) and subsequent taxonomic profiling of the protein coding genes suggests that the microbial processes associated with this particular microbiome are dominated by members of the *Proteobacteria*, *Firmicutes*, *Bacteroidetes*, *Chloroflexi* and *Euryarchaeota*. Members of the *Oceanospirillales*, a bacterial order that recruited more than 60% of the genes from the DWH oil plume metagenome [14], recruited only a small fraction (<2%) of the genes from the SBC metagenome, which suggests that *Oceanospirillales* might play a less significant role in the microbially mediated hydrocarbon conversion within the SBC seep oil compared to the DWH plume oil, which had an average oxygen saturation of 59% [4]. Oxygen depletion in SBC sediment has been reported previously [62] and we hypothesize that the distinct taxonomic fingerprint of the genes assembled from the SBC seep oil and DWH oil plume metagenome data is caused in part by the different concentrations of oxygen within these oils. This hypothesis is supported by recent findings by Kimes et al [66] that showed that *Oceanospirillales* contributed only a small fraction to the overall microbiome associated with cores collected from low oxygen sediments in the GoM. The hypothesis that the SBC seep oil contains low concentrations of oxygen and thus facilitates anaerobic processes is supported by the results from our functional gene analysis of the SBC seep oil metagenome, which revealed the presence of the genes essential for anaerobic methane oxidation, and the findings that members of the anaerobic methanotrophic archaea comprise the majority of the archaeal genes within the SBC seep oil metagenome. Taking these findings into consideration, it appears plausible that the taxonomic and functional make-up of the metagenome associated with the SBC seep oil and the DWH plume oil depends rather on the oxygen saturation of the oil then its geographical origin and that the metabolic capability of the associated microbiome might be dynamic. However, further studies are necessary to obtain a better understanding of the biological processes that are associated with these hydrocarbons and their microbially mediated degradation process.

The metagenome from natural oil that seeps into the SBC and the metagenome associated with the oil plume that formed in the aftermath of the DWH blowout are publicly accessible for further analysis at IMG/M. This provides a unique opportunity to study the metabolic profile of a hydrocarbon degrading community from the SBC and to infer the metabolic differences between microbial communities associated with natural hydrocarbons that enter the marine ecosystem at different geographical locations.
